# MiR-214 and N-ras regulatory loop suppresses rhabdomyosarcoma cell growth and xenograft tumorigenesis

**DOI:** 10.18632/oncotarget.1855

**Published:** 2014-03-25

**Authors:** Hui-jie Huang, Jun Liu, Hu Hua, San-en Li, Jin Zhao, Shen Yue, Ting-ting Yu, Yu-cui Jin, Steven Y. Cheng

**Affiliations:** ^1^ Department of Developmental Genetics, School of Basic Medical Sciences, Nanjing Medical University, 140 Hanzhong Road, Nanjing, Jiangsu 210029, China; ^2^ MOE Key Laboratory of Model Animal for Disease Study, Model Animal Research Center, Nanjing University, 12 Xuefu Road, Pukou District, Nanjing, Jiangsu 210063, China

**Keywords:** rhabdomyosarcoma, miR-214, N-ras, RD cells, tumor suppressor

## Abstract

Rhabdomyosarcoma (RMS) is a childhood malignant soft tissue cancer that is derived from myogenic progenitors trapped in a permanent mode of growth. Here, we report that miR-214 is markedly down-regulated in human RMS cell lines. Although not required for embryogenesis in mice, miR-214 suppresses mouse embryonic fibroblast (MEF) proliferation. When re-introduced into RD cells, a line of human embryonal RMS cells, miR-214 showed inhibition of tumor cell growth, induction of myogenic differentiation and apoptosis, as well as suppression of colony formation and xenograft tumorigenesis. We show that in the absence of miR-214, expression of proto-oncogene N-ras is markedly elevated in miR-214^−/−^ MEFs, and manipulations of miR-214 levels using microRNA mimics or inhibitor in RD cells reciprocally altered N-ras expression. We further demonstrate that forced expression of N-ras from a cDNA that lacks its 3'-untranslated region neutralized the pro-myogenic and anti-proliferative activities of miR-214. Finally, we show that N-ras is a conserved target of miR-214 in its suppression of xenograft tumor growth, and N-ras expression is up-regulated in xenograft tumor models as well as actual human RMS tissue sections. Taken together, these data indicate that miR-214 is a bona fide suppressor of human RMS tumorigensis.

## INTRODUCTION

Rhabdomyosaroma (RMS) is a rare form of soft tissue cancer affecting muscles throughout the body [1, 2]. Histologically, RMS is broadly divided into embryonal and alveolar subtypes, which arise in children from age 1 to 5 and older ones, respectively. Some rare forms of RMS also occur in the adult, and are generally more malignant [3]. The alveolar RMS is known for a characteristic chromosomal rearrangement [t(2;13) (q35;q14)] that fuses the forkhead homologous group gene FKHR on chromosome 13 to the paired box gene PAX3 on chromosome 2, and a variant of this [t(1;13) (p36;q14)], to PAX7 on chromosome 1 [4-7]. The molecular pathogenesis of the embryonal RMS is less clear, although allelic loss at chromosome 11p15, a locus overlapping with a Beckwith-Wiedemann syndrome critical region [8], was reported [9], and aberrant activation of the Sonic Hedgehog pathway was linked to the embryonal RMS in mice [10, 11]. Both forms of RMS are thought to be derived from myogenic progenitors as the consequence of impaired differentiation due to genetic lesions [12-14]. Current conventional treatments for RMS show varying prognostic outcomes, depending on the location of the tumor [1]. Further studies are required for a better understanding of the RMS etiology and the development of targeted treatment strategies.

During development, a network of transcription factors orchestrates the expression of genes that program muscle growth, differentiation, and contractility [15, 16]. In addition to the protein-coding genes, recent studies have revealed a collection of microRNAs that play very important roles in regulating muscle development as well as physiological functions [17-19]. Some of these microRNAs that exhibit specific patterns of muscle expression are dubbed “myomiRs”; these include members of the bicistronic miR-1/133a and miR206/133b families [20], and a group of microRNAs, namely miR-208, miR-208b, and miR-499, that are embedded in genes encoding the myosin heavy chain [21]. Other microRNAs with important muscle functions such as miR-29 [22] and miR-181 [23] can have a broad expression pattern in many tissues [18, 19]. In cardiac and skeletal muscles, myogenic transcription factors MyoD, MEF2, and SRF drive the expression of miR1/206/133 clusters directly through upstream or intronic cis-regulatory elements [17]. These microRNAs in turn target HDAC4, HAND2, and a wide variety of other key factors that regulate muscle cell differentiation and physiological functions [18]. Aggregated studies from the past decade indicate that although microRNA sequences are highly conserved in evolution, their functions do not appear to contribute significantly to embryonic development; instead, microRNAs play very important roles in orchestrating cellular responses to both physiological and pathological stress [24]. Since oncogenic transformation imposes enormous stress to essentially every cellular process, the microRNA regulatory network is frequently altered in cancer [25]. Indeed, dysregulation of microRNAs in RMS is a wide spread phenomenon for many specific microRNAs [26], and re-expression of miR-1/133a, miR-206, and miR-29 in RMS cells have been shown to induce myogenic differentiation and block xenograft tumorigenesis [22, 27].

MiR-214 is a ubiquitously expressed microRNA with important muscle function. The myogenic function of miR-214 was first reported in zebrafish [28], in which its downregulation by morpholino-mediated RNA silencing led to a loss of the slow muscle cells due to interruption of normal Hedgehog signaling. Although not required for embryonic development in mammals [29, 30], miR-214 is capable of regulating the differentiation of myogenic progenitor cells through many of its evolutionarily conserved targets and by many mechanisms. In mouse C2C12 myoblasts, miR-214 was reported to form a negative feedback loop with a polycomb group component Ezh2 that controls the expression of miR-214 as well as myogenic transcription factors MyoD and Myogenin through epigenetic modifications of the chromatin structure [31]. MiR-214 was also noted to promote cell cycle exit, a prerequisite of cell differentiation [32], and through global gene expression profiling, N*-ras* was identified as a target that mediates the miR-214 myogenic function [33]. Activating mutations in N*-ras* and K*-ras* were identified in human embryonal RMS samples many years ago [34] and forced expression of N*-*ras in human normal skeletal muscle cells [35] as well as in zebrafish [36] both led to the establishment of RMS models. Thus, miR-214 likely possesses an anti-tumor function in suppressing RMS tumorigenesis through N-ras.

Here, we show that miR-214 expression is significantly down-regulated in a number of RMS cell lines relative to normal human skeletal muscles and fibroblasts. Using chemically synthesized microRNA mimics and precursor microRNA (pre-miRNA) expression vectors, we demonstrate that miR-214 is a potent growth inhibitor and a suppressor of RMS tumorigenesis, acting on human N*-ras,* a conserved target of the miR-214 myogenic and tumor suppressor functions.

## RESULTS

### MiR-214 inhibits embryonic cell proliferation

**Figure 1:**
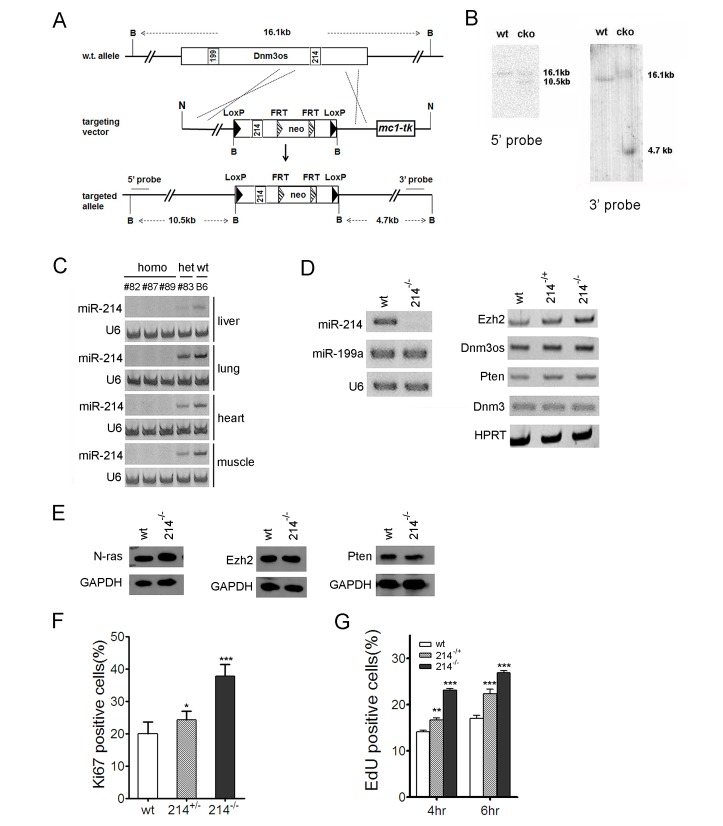
miR-214 inhibits the proliferation of murine embryonic fibroblasts (A) Schematic representation of the miR-214 genomic locus and the conditional knockout targeting construct. B: BamHI, N: NotI. (B) Southern blot analyses of the BamHI restricted DNA. Positions of the 5' and 3' probes were shown in (A). (C) Stem-loop RT-PCR detection of miR-214 in the liver, lung, heart, and muscle of homozygous and heterozygous miR-214^−^ as well as the B6 control mice. (D) Stem-loop RT-PCR detection of miR-214 and miR-199a2 (left), as well as regular PCR detection of Dnm3os, Dnm3, N-ras, Ezh2, and Pten expression in B6 control, miR-214^−/+^, and miR-214^−/−^ MEFs. U6 snRNA and HPRT were used as loading controls in (C) and (D). (E) Western analyses of N-ras, Ezh2, and Pten in miR-214^−/−^ and B6 control MEFs. (F) Quantifications of ki67 staining and (G) Edu incorporation in B6 control, miR-214^−/+^, and miR-214^−/−^ MEFs. The fluorescence images used for these graphs are shown in supplementary sFig.3 and sFig.4, respectively. Asterisks here and in all subsequent figures denote P values computed based on one-way ANOVA unless noted otherwise. * = p<0.05, ** = p<0.01, and *** = p<0.001.

MiR-214 is encoded along with miR-199a in a 7.8 kb bi-cistronic primary microRNA transcription unit, Dynamin 3 opposite strand (Dnm3os), which is embedded on the reverse strand in an intron of the Dynamin 3 (Dnm3) gene [33]. To investigate its physiological function, we generated a conditional mouse miR-214 knockout allele (cko) by inserting two LoxP sites immediately flanking the pre-miR-214 sequence (Fig.1A). Southern blot analyses of BamHI restricted genomic DNA confirmed the insertion of the LoxP sites and the neomycin selection marker (Fig.1B). Germline deletion of the miR-214 locus was achieved by crossing miR-214^cko^ to EIIa-cre driver mice that express the cre recombinase ubiquitously. Homozygous miR-214^−/−^ mice were born healthy at the expected Mendelian ratio and exhibited no overt developmental abnormally. These animals also aged normally with an average rate of cancer expected for the B6 strain background. Examination of the liver, lung, heart, and muscle by stem-loop PCR detection showed a robust miR-214 expression in the control B6 mice, but miR-214 is absent from those tissues in the homozygously deleted mice or at a reduced level in the heterozygotes (Fig.1C). To determine if miR-214 plays a role in the homeostatic maintenance of the muscle function in the adult, we created the muscle injury model in miR-214^−/−^ and the control B6 mice by cardiotoxin III injection in the tibia calf [37]. Histological examination indicated that the injury sites in both types of mice were completely repaired after two weeks of recovery, although the repair process in miR-214^−/−^ mice was slightly delayed as indicated by the lower density of regenerating (centralized) nuclei in the injury areas compared to that of the wt control mice (Supplementary sFig.1). Since miR-214 was reported to be one of the up-regulated microRNAs during cardiac hypertrophy [38], we also examined the role of miR-214 in the adult heart by the transverse aortic constriction procedure [39]. Once again, no statistic significant difference was observed in a range of parameters between miR-214^−/−^ and the control B6 mice (Supplementary sFig.2).

To determine if miR-214 exerts any physiological control on cell growth and differentiation, we isolated the primary murine fibroblasts (MEFs) from miR-214^−/−^ and control B6 embryos. As expected, genomic ablation of miR-214 did not alter the expression of the cistronic miR-199a or Dnm3os, the noncoding primary RNA (Fig.1D). We also did not detect any change in the expression of the host gene Dnm3 (Fig.1D), suggesting that Dnm3 expression is not affected by the intronic deletion. In contrast, the levels of N*-*ras mRNA transcript and protein increased markedly in miR-214^−/−^ MEFs (Fig.1D, 1E), in keeping with our previous observation in C2C12 cells [33]. However, we were unable to detect any changes in either mRNA (Fig.1D) or protein levels (Fig.1E) of Ezh2 [31] and Pten [40] in miR-214^−/−^ MEFs compared to the B6 control. Nevertheless, immunofluorescence staining of Ki67 indicated a progressive increase in the rate of ribosomal RNA transcription after removing one (heterozygotes) or both (homozygotes) alleles of miR-214 (Fig.1F, supplementary sFig.3), suggesting that miR-214 inhibits cell proliferation. Indeed, direct quantification of cell growth by EdU incorporation assays showed approximately 25% and 65% increase in the growth rates of the heterozygous and homozygous miR-214^−^ MEFs compared to the B6 control, respectively (Fig.1G, supplementary sFig.4). Thus, although miR-214 is not essential for embryonic development and loss of miR-214 by itself is not sufficient to cause cancer in mice, it normally exerts a negative control on cell growth.

### Dysregulation of miR-214 in RMS cell lines correlates with its growth inhibitory property

**Figure 2:**
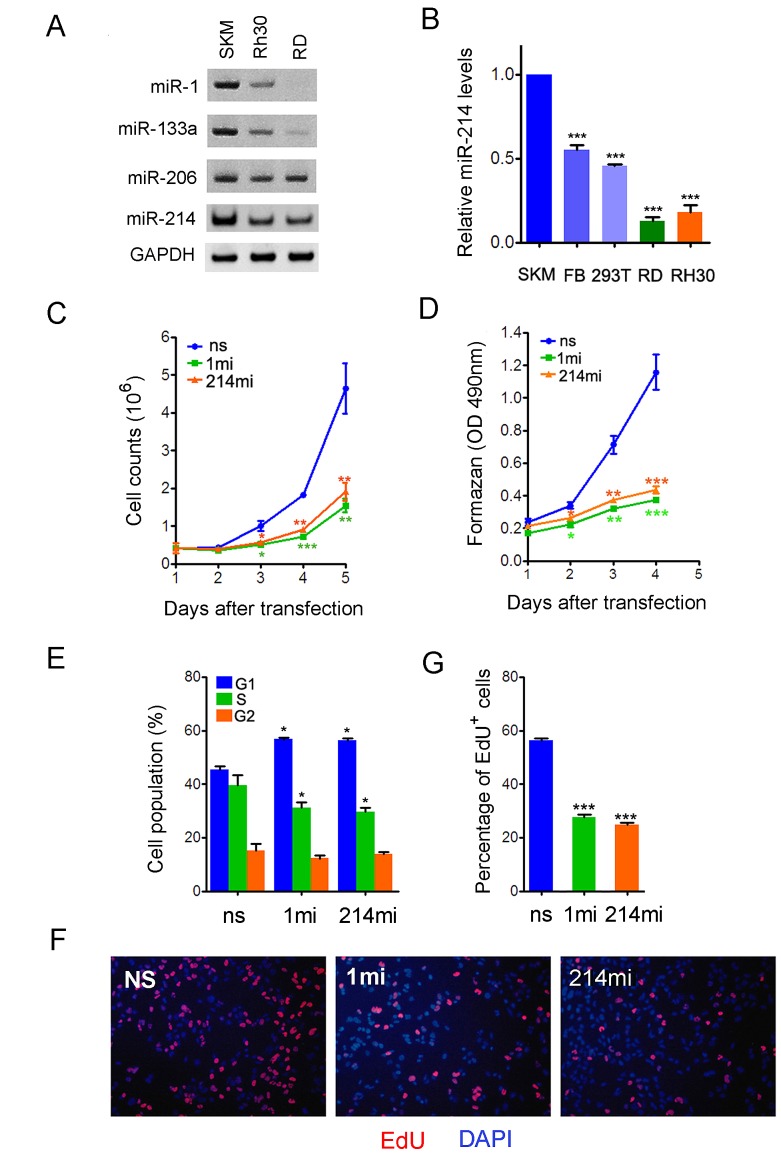
miR-214 is a suppressor of human RMS cell growth (A) RT-PCR detection of miR-1, miR-133a, and miR-214 in RD and Rh30 cells, as well as in normal skeletal muscles (SKM). U6 snRNA was used as loading control. (B) qPCR quantification of miR-214 expression with a specific TaqMan probe. The data were compiled from three rounds of experiments and are presented as mean ± standard deviation. SKM, extraocular muscle from the eye. FB, primary fore skin fibroblasts. (C) Cell counts and (D) MTT assays for RD cells transiently transfected with nonsilencing control nucleotides (ns), miR-1 mimic (1mi), or miR-214mimic (214mi). (E) FACS analysis of the cell cycle profile in RD cells transiently transfected with ns, 1mi, or 214mi. 10,000 cells were sorted in each sample and the cells were collected 48 hours after transfection and stained with propidium iodide. (F) Fluorescence images and (G) percentage quantification of Edu incorporation assays as in (E). The cells were assayed in 24-well plates.

In light of miR-214 roles in promoting myogenic differentiation and cell growth control, we speculated that it might possess a tumor suppressor function. Since RMS is a cancer of dysregulated myogenic precursors, we sought to determine if miR-214 regulates RMS cell growth. To address this possibility, we examined miR-214 expression in two human RMS cell lines, RD and Rh30, which are derived from tumors of the embryonal and alveolar origin, respectively. Using the stem-loop RT-PCR and by comparing to endogenous U6 snRNA, we observed a noticeable reduction of miR-214 expression in both RD and Rh30 cells relative to its level in the normal human skeletal muscle (Fig.2A). The reduction in miR-214 expression was even more pronounced when RNA levels were examined using the Taqman real time PCR (Fig.2B). We further compared miR-214 expression in RD and Rh30 cells to that in primary human fibroblasts and HEK293 cells and found that tumor miR-214 levels were also reduced (Fig.2B), suggesting that the reduction of miR-214 expression in RMS cells cannot be simply attributed to adaptation to cell culture, rather it is probably an intrinsic property of cancer cells. The levels of muscle specific miR-1 and miR-133a also decreased in RD and Rh30 cells in accordance with the oncogenic transformation, although the level of miR-206 did not show significant change (Fig.2A).

To test directly if dysregulation of miR-214 influences RMS cell growth, we transfected RD cells with microRNA mimics (mi) or a non-silencing control oligonucleotide (ns), and measured their effects on cell proliferation by a number of assays. Relative to those transfected by ns, RD cells that received miR-214mi grew much slower by daily cell counts (Fig.2C) and had reduced rate of metabolism as evident by quantification using tetrazolium dye, 3-(4,5-dimethylthiazol-2-yl)-2,5-diphenyl tetrazolium bromide (MTT) (Fig.2D). The extent of growth inhibition exerted by miR-214mi was comparable to that by the mimic of miR-1, which is known to suppress RMS cell growth [41]. We also tested the anti-proliferative activity of miR-214 in prostate tumor lines, DU-145 and PC3, by over-expressing miR-214 from a MSCV-based viral vector P2GM [42] and observed similar growth retardation (Supplementary sFig.5). Flow cytometry analysis indicated that miR-1mi and miR-214mi increased the fraction of RD cells in the G1 phase from 45.5% in the ns control to around 56.4% in the mimics treatment groups (Fig.2E). Consistent with the expansion of the G1 phase population, the mitotic indices of the RD cells transfected by miR-1mi and miR-214mi also decreased significantly (Fig.2F and 2G). Taken together, these results indicate that miR-214 is a growth inhibitor of different types of tumor cells.

### miR-214 promotes apoptosis and myogenic differentiation of RD cells

**Figure 3:**
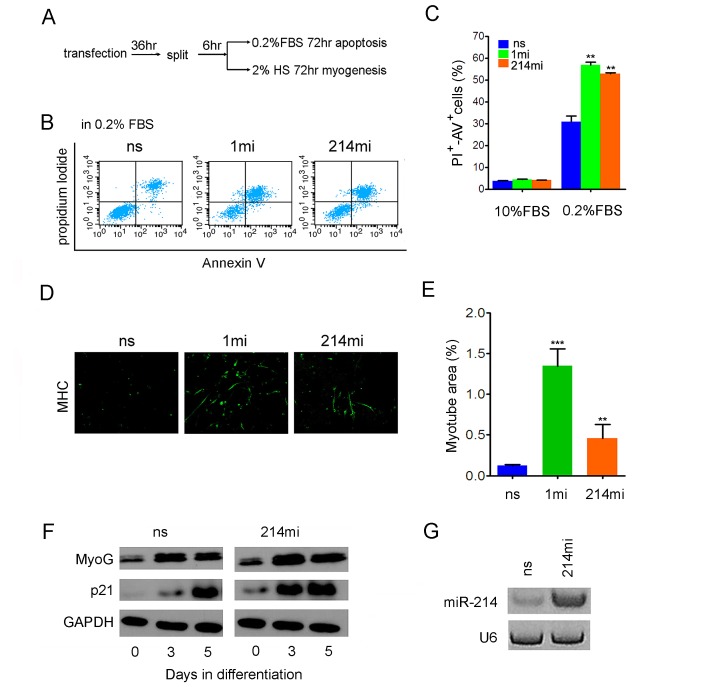
miR-214 promotes apoptosis and myogenic differentiation of RD cells (A) diagram of experimental flow. RD cells were transiently transfected with ns, 1mi, or 214mi in 60 mm petri dishes for 36 hours before they were split into 6-well plates. For apoptosis assays (B) and (C), the cells were maintained in DMEM supplemented with 0.2% FBS and processed for apoptosis assay 3 days later by Annexin V and PI double staining and FACS analysis. For induction of myogenic differentiation, the cells in 6-well plates were incubated in differentiation medium containing 2% horse serum for 5 days and then stained with anti-MHC. (D) Image and (E) quantification of myotube areas using Image J software. Original objective magnification is 20x. (F) Western analyses of p21 and MyoG levels in RD cells transiently transfected with miR-214mi and the ns control for time as indicated. (G) Stem-loop RT-PCR detection of miR-214 in RD cells five days after transfection.

Since RMS arises from oncogenic transformation of myogenic progenitor cells, blocking RMS cell growth should induce apoptosis or differentiation [43]. To further characterize the tumor suppression properties of miR-214, we examined the ability of RD cells to undergo apoptosis and differentiation after they received miR-214mi or controls through transfection. After transfection with microRNA mimics, apoptosis was induced by maintaining the cells in DMEM supplemented with 0.2% FBS for 72 hours and quantified using Annexin V and propidium iodide (PI) double staining. Flow cytometry analyses indicated that after serum depletion for 3 days, about 32% of RD cells transfected with the ns control became double positive for Annexin V and PI, an indication of late stage of cell death [44], but this percentage increased to 56.9% and 52.8% when the cells were transfected with miR-1mi and miR-214, respectively (Fig.3B and 3C). In the presence of 10% FBS, RD cells did not undergo apoptosis and neither miR-1 nor miR-214 were able to induce such (Fig.3C). Once again, miR-214 is not a sufficient inducer of apoptosis on its own. In keeping with their pro-apoptotic activity, both miR-1mi and miR-214mi induced myogenic differentiation of transfected RD cells as measured by immunofluorescence staining of myosin heavy chain (MHC) (Fig.3D and 3E), although the induction by miR-1mi was more pronounced. Consistent with MHC staining, the onset of cell cycle inhibitor p21^ink4a^ up-regulation, a prerequisite of myogenic differentiation [45, 46], advanced by 3 days in RD cells that received miR-214mi (Fig.3F). During the 5-day span of differentiation, total level of miR-214 in the transfected RD cells was maintained at a higher level than the ns control by the mimic (Fig.3G), suggesting a causal relationship between miR-214 overexpression and accelerated exit from the mitosis.

### miR-214 inhibits colony formation and xenograft tumor growth

**Figure 4:**
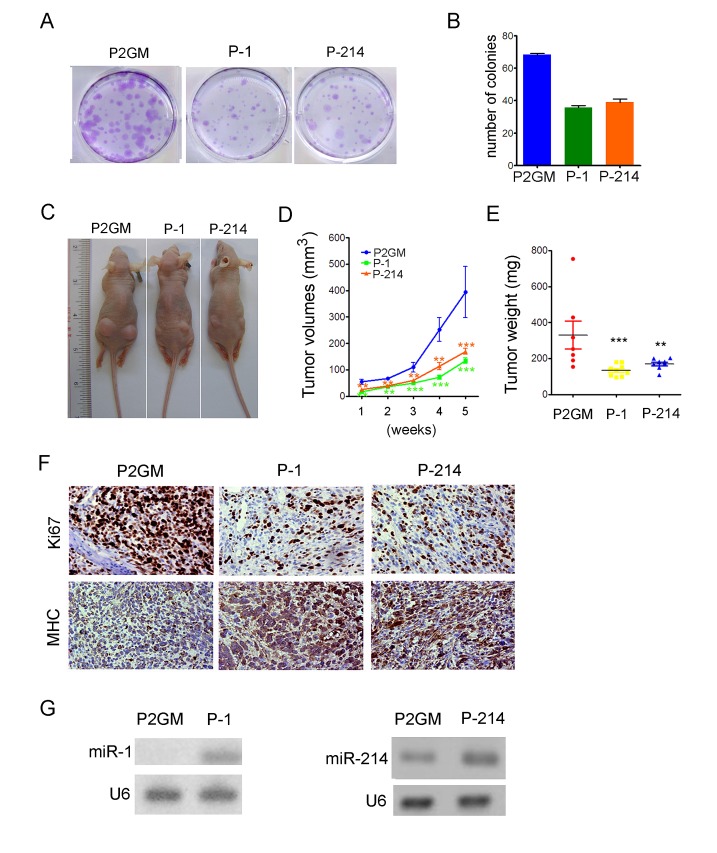
miR-214 suppressed colony formation and xenograft tumorigenesis (A) 500 stable RD cells carrying constitutive P2Gm vector, P2Gm-miR-1, or P2Gm-miR-214 were cultured in 60 mm petri dishes in the presence of 10 μg/ml puromycin for 14 days. The plates were then fixed and stained with crystal violet for colony counting. (B) Quantification of (A). The experiments were repeated three times and each data point was done in duplication. (C) Images of tumor-bearing nude mice taken after the animals were euthanized at the end of a 6-week observation period. (D) Weekly measurements of average tumor volumes, and (E) the range of individual terminal tumor weights were plotted. (F) IHC staining of xenograft tumor sections for MHC and the proliferative antigen Ki67. Original objective magnification is 40x. (G) Stem-loop RT-PCR detection of miR-1 and miR-214 levels in terminal xenograft tumors.

To further demonstrate its tumor suppression function, we asked if forced expression of miR-214 inhibits the ability of RD cells to form anchorage-dependent or independent foci in culture or xenograft tumors in nude mice. For these purposes, we generated stable RD cells expressing pre-miR-214 or pre-miR-1 from the constitutive P2GM vector. After plating approximately 500 stably transfected cells in a 60 mm petri dish and culturing for 14 days, we observed about 68 colonies of RD cells carrying the P2GM vector, and below 40 colonies of RD cells expressing either miR-1 or miR-214 (Fig.4A and 4B). Moreover, the average sizes of miR-1 and miR-214-expressing colonies were much smaller than that of the vector cells (Fig.4A). Similar results were obtained with RD cells transiently transfected with microRNA mimics (Supplementary sFig.6A, 6B). When assayed for anchorage-independent growth in top agar plates, stable RD cells expressing pre-miR-1 or pre-miR-214 also formed fewer foci than the P2GM RD cells (Supplementary sFig.6C, 6D), albeit miR-1 exhibited more potent activity in suppressing the anchorage-independent colony formation than miR-214. For xenograft tumor growth experiment, we injected approximately 8 million stable RD cells subcutaneously into two bilateral sites on the lower back of 6 weeks old BALB/c nude mice. The tumors were measured weekly for 5 consecutive weeks, and each tumor was individually weighed after the mice were euthanized. Compared to the vector-bearing control RD cells, those that expressed pre-miR-1 or pre-miR-214 grew much slower (Fig.4C, and 4D), and reached to smaller terminal sizes (Fig.4E). On histological sections, xenograft tumors expressing pre-miR-1 or pre-miR-214 showed decreased staining for Ki67 but increased staining for MHC (Fig.4F), suggesting a benign growth relative to the vector-bearing tumors. Stem-loop RT-PCR confirmed the ectopic expression of miR-1 and miR-214 in their respective tumors (Fig.4G).

### miR-214 inhibits RD cell growth and differentiation through N-ras

**Figure 5:**
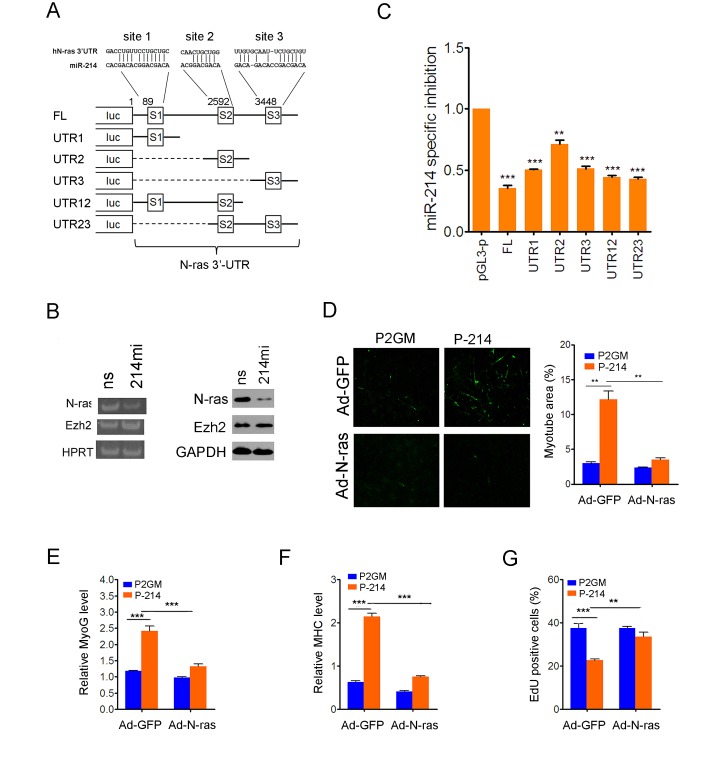
Human N-ras is a conserved tumor suppression target of miR-214 (A) Predicted miR-214 recognition sites in the 3'-UTR of human N-ras and schematic representation of human N-ras 3'UTR reporter constructs. (B) RT-PCR (left) and Western blot (right) analyses of N-ras and Ezh2 in RD cells transfected with miR-214mi and the ns control. (C) Luciferase reporter assays for differential inhibition by various 3'-UTR sequences of human N-ras in P2GM and P-214 stable RD cells. The miR-214 specific inhibition is defined in the text. The luciferase activities were quantified 48 hours after transfection and each data point represents the average of three independent experiments done in duplicates. P-values were calculated based on t-tests. (D) Immunofluorescence staining of MHC and quantification of myotube areas thereof in RD cells differentiated in 6-well plates for 5 days. Infection by Ad-N-ras and Ad-GFP control was carried out prior to the induction of differentiation. Myotube area was calculated from 6 randomly chose fields in each of three separate wells. Each data point represents the average from three repeated experiments. Original objective magnification is 20x. (E) SYBR green qPCR quantification of MyoG and (F) MHC in the above differentiated RD cells at the end of 5 days. (G) Percentage of EdU incorporation in stable RD cells 12 hours after infection with Ad-N-ras or the control Ad-GFP.

Mouse N*-ras* contains two miR-214 recognition sites in its 3'-UTR, whereas human N*-ras* has one site that matches to the 7-nucleotide seed sequence of miR-214 and two imperfect sites (Fig.5A). Semi-quantitative PCR detected a noticeable decrease in human N*-ras* expression in RD cells following transfection of miR-214mi (Fig.5B, left), and transfection of RD cells with miR-214mi also reduced the level of N*-*ras protein (Fig.5B, right), but again no change was seen in either the mRNA or protein levels of Ezh2 (Fig.5B). To determine if human N*-ras* is also a direct target of miR-214-mediated translational regulation, we cloned the 3'-UTR of human N*-ras* in the pGL-3p vector and made various mutant constructs lacking miR-214 recognition sites (Fig.5A). We then compared the luciferase activities of these constructs in the vector control P2GM and pre-miR-214-expressing stable RD cells, and calculated miR-214-specific inhibition, which is defined as the ratio of luciferase activities between these two pools of RD cells normalized for the value elicited from the pGL3-p vector in the P2GM carrying RD cells. Prior to the calculation, all luciferase activity values were adjusted against co-transfected renilla luciferase for controlling transfection efficiency. The results indicated that the full length 3'UTR of N-ras afforded a 60% inhibition of the pGL3-p luciferase activity that could be attributed to the miR-214 over-expression in the P-214 stable RD cells (Fig.5C). Each of the three miR-214 recognition sites exhibited inhibitory activity with site 1 showing the strongest while site 2 the weakest (Fig.5C). To further demonstrate that human N-ras is a major target gene that mediates the tumor suppressor activity of miR-214 in RD cells, we infected the P2GM and P-214 stable RD cells with adenoviruses that express N-ras from a microRNA-resistant, 3'-UTR-less cDNA. After switching the cells to differentiation medium and incubation for 5 days, we found that forced expression of N-ras neutralized the pro-myogenic effect of miR-214 in RD cells, which was determined by immunofluorescence staining of MHC (Fig.5D), and RT-PCR detection of myogenin (Fig.5E) and MHC (Fig.5F) mRNAs [33]. Forced expression of N-ras also reversed the anti-proliferative effect of miR-214 (Fig.5G). Thus, N*-ras* is a conserved target of miR-214 in human cells as well.

### Up-regulation of N-ras is associated with RMS tumorigenesis

**Figure 6:**
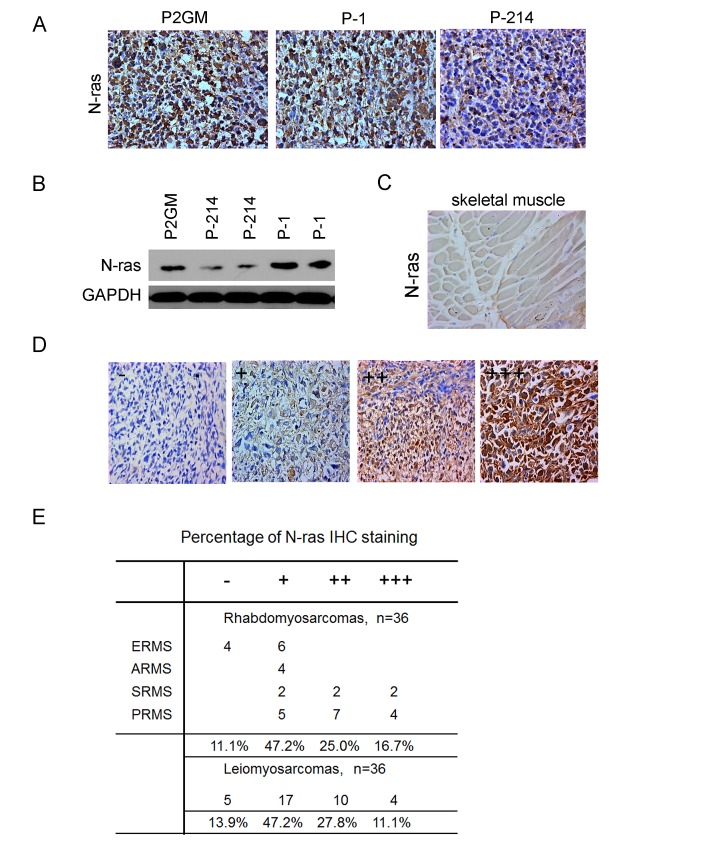
Up-regulation of N-ras in xenograft tumors and primary human RMS tumors (A) IHC staining of N-ras in xenograft tumors derived from RD stable cells carrying P2GM, P2GM-miR-1, and P2GM-miR-214 constructs. (B) Western analyses of N-ras from each line of P2GM, P-214, and P-1 stable RD cells and their xenograft tumor derivative. (C) IHC staining of N-ras in the normal skeletal muscle control in the human primary RMS tumor array slide and (D) the tumors. The slide was read twice by two individuals and the level of N-ras expression was stratified according to the matrix defined here. (E) Quantification of N-ras levels in leimyosarcoma and sub-rhabdomyosarcoma categories in the tumor array slide.

Having demonstrated the growth inhibitory function of miR-214 through N*-ras*, we sought to determine if this regulatory loop actually is associated with tumorigenesis. For this purpose, we first examined the level of N*-*ras expression in our xenograft tumor samples by IHC staining, which detected a robust N-ras expression in P2GM or P-1 xenograft tumors (Fig.6A). In contrast, N-ras expression was dramatically lower in P-214 tumors (Fig.6A). Consistent with the IHC results, Western blot analyses showed a marked up-regulation of N-ras in xenograft tumors formed by all three lines of stable RD cells compared to their parental cells, even though N-ras expression was lower in P-214 RD cells and the tumors than in the P2GM and P-1 lines of RD cells and the tumors, respectively (Fig.6B). So, despite both miR-1 and miR-214 are able to induce RD cells to undergo myogenic differentiation (Fig.3D, 3E) and suppress their tumorigenic activities (Fig.4A-4E), only miR-214 reached these outcomes through blocking N-ras. Since siRNA-mediated knockdown of N-ras in RD cells drastically enhanced their ability to differentiate along the myogenic lineage and retarded their proliferation (Supplementary sFig.7), N-ras had to play an active role in RD cell regulation. This observation likely explains why miR-214 is effective in suppressing RMS tumorigenesis. To determine if N*-*ras contributes to tumorigenesis in actual human RMS samples, we then examined its expression in a human tumor tissue array. This array slide contains both leiomyosarcoma (LMS) and RMS sections, as well as normal muscle controls. In the skeletal muscle control, N-ras expression was barely detectable (Fig.6C), but varying levels of N*-*ras, stratified as absent (-), low (+), moderate (++), and high (+++) according to the extent of IHC staining, were seen in tumor sections (Fig.6D). Out of the 36 RMS, 17 exhibited low, 9 moderate, and 6 high levels of N*-*ras (Fig.6E). This translates to close to 90% RMS samples being positive for N*-*ras expression. Similar distribution of N*-*ras expression was seen among LMS sections (Fig.6E), and two other adult and more malignant forms of RMS, sclerosing and pleiomorphic, exhibited even higher levels of N*-*ras expression (Fig.6E). These data strongly suggest a causal relationship between N*-*ras up-regulation and RMS tumorigenesis.

**Figure 7:**
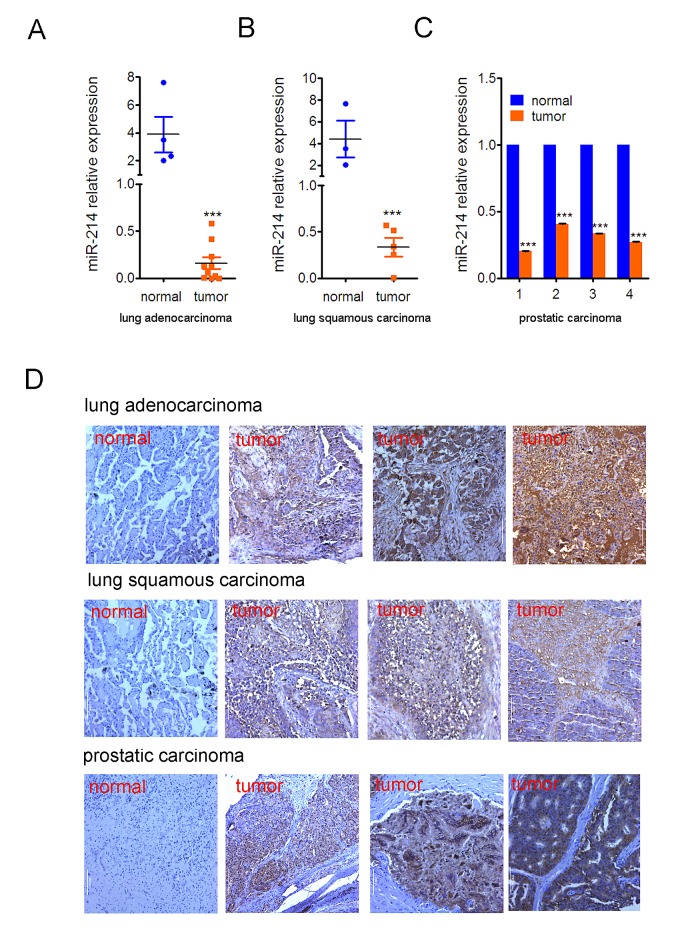
Inverse correlation of miR-214 and N-ras expression in lung and prostate cancers RT-qPCR quantification of miR-214 expression in human (A) lung adenocarcinomas, (B) lung squamous carcinomas, and (C) prostatic carcinomas. Pair-wise comparison of prostatic carcinomas and their matching normal tissue samples were used in (C). Bars denote standard deviation, and Student T test was used for assessing P values. ***, P<0.001. (D) IHC staining of N-ras. A representative staining of the normal tissue and three tumor samples were shown for each type of cancer.

To determine if the miR-214 and N-ras regulatory loop also applies to other types of tumors, we examined the expression of these two genes in adenocarcinomas and squamous carcinomas of the lung as well as prostatic carcinomas by qPCR and IHC staining, respectively. The results indicated that expression of miR-214 was drastically down-regulated in both types of lung cancers as compared to normal lung tissues (Fig.7A, 7B). The same was true in a pair-wise comparison of prostatic carcinomas and matching normal tissues (Fig.7C). In contrast, IHC staining showed a clear increase in N-ras expression in all three types of tumors (Fig.7D).

## DISCUSSION

Although a rare form of cancer in children, RMS draws research interest not only for insights into innovative treatment strategies, but also for basic knowledge of muscle biology, because causal genetic lesions often expose key regulatory mechanisms of muscle growth and differentiation. In this study, we investigated the roles of miR-214 in suppressing RMS cell growth and xenograft tumor formation. Our results indicate that miR-214 exerts its tumor suppressor function by targeting proto-oncogene N*-ras* in both mouse and human cells. The tumor suppressor function of miR-214 is directly related to its normal role in promoting cell cycle exit, a prerequisite to myogenic differentiation. This mechanism is particularly relevant to RMS since it is a form of cancer originated from myogenic progenitors that were trapped in an indefinite division mode. Like all microRNAs, miR-214 either suppresses the protein product translation or induces messenger RNA degradation of a wide range of target genes by forming imperfect base-pairing between its seed sequence and recognition sequences in the mRNA target 3'-UTR [47]. We previously identified N*-ras* as a target of miR-214 that mediates its myogenic function in mouse myoblasts, and found the miR-214 recognition sequences in both mouse and human N*-ras* [33], thus the sequence basis for the functional conservation in these two species. Our current investigation further extended miR-214 function to tumor suppression and revealed a likely causal correlation between marked up-regulation of N*-ras* and human RMS tumorigenesis. Several recent studies identified other genes that are regulated by miR-214, such as Ezh2 and Pten [26, 31, 40]; however, we did not detect any change in these two protein levels in miR-214^−/−^ MEFs. Perhaps those regulatory loops are required for under different physiological conditions. In contrast, both the messenger RNA and protein levels of N*-ras* increased dramatically when miR-214 was lost completely. These results argue that N*-ras* is likely a major target of miR-214, and call for further scrutiny of miR-214 expression in primary human RMS samples.

Given their importance in gene regulation, functions of many microRNAs have been examined for the involvement in RMS tumorigenesis [26]. Several studies reported decreased levels of miR-1, miR-206, and miR-133a in primary RMS tumor samples and cell lines [27, 41]. Re-expression of these muscle-specific microRNAs was shown to inhibit cell growth, promote differentiation, and retard xenograft tumor formation [27, 41]. We compared the function of miR-214 to miR-1 and found its ability to do all above was comparable. So, despite having a different set of targets and regulating muscle differentiation by a different mechanism, miR-214 is also a potent suppressor of RMS cell growth. These microRNAs form a complex regulatory network exerting a tight control over the myogenic differentiation and muscle function through fine-tuning the expression of underlying protein encoding genes. It is likely that during tumorigenesis the entire microRNA network was rebalanced for the maximum gain of advantages towards acquiring cell growth and other hallmarks of cancer. MiR-214 is part of this network, although its function is not required during embryonic development as miR-214 null embryos were carried to full term and the pups grew healthy and fertile [29, 30]. This network has many regulatory nodes responding and reacting to different environmental stimuli. The node defined by miR-214 likely reacts to stress cues as it has been demonstrated to protect the mouse heart from ischemic injury by controlling Ca^2+^ overload and death [29]. During the process of tumorigenesis, pre-cancerous cells endure enormous selective pressure to reorganize their metabolic program and miR-214 becomes engaged to counter tumor growth.

Many genes that show activities of inhibiting tumor cell growth under cell culture conditions are not operative in actual tumors. However, our data on the contrary showed that miR-214 is engaged in tumor suppression in vivo. In xenograft tumors formed by the MSCV-P2GM vector-bearing RD cells that did not express miR-214, N*-ras* exhibited robust expression. This observation is consistent with published reports that detected activating N*-ras* and K*-ras* mutations in human embryonal RMS samples [34] and induction of RMS models by force expression of activating N*-ras* in human normal skeletal muscle cells [35] and zebrafish [36]. Indeed, we also found up-regulation of N-ras in primary human RMS samples and xenograft RMS tumor models (Fig. 6B-E). Thus, the mitogenic signaling pathway initiated by Ras activation is critical to RMS tumorigenesis. When re-introduced back into RD cells, miR-214 ostensibly blunted N*-ras* expression and suppressed the xenogaft tumor growth (Fig.6A). These findings strongly support the relevance of miR-214 and N-*ras* regulatory loop in RMS etiology. For these reasons, miR-214 is likely a good candidate for the development of an anti-RMS therapeutic antagomiR.

## MATERIALS AND METHODS

### Cell culture and plasmids construction

Human RMS cells RD and RH30, prostate cancer cells DU-145 and PC-3, primary foreskin fibroblasts, HEK293 cells, and mouse MEFs were maintained in DMEM supplemented with 10% fetal bovine serum, 100 U/ml penicillin and 100 μg/ml streptomycin at 37°C with 5% CO_2_. Human genomic DNA fragments containing pre-miR-1 or pre-miR-214 sequences were amplified by PCR and inserted at the NotI and PmeI site in the MSCV-P2Gm vector. Full length or fragments of the N-ras 3'-UTR containing miR-214 recognition sequences were inserted behind the luciferase coding sequence at the XbaI site in the pGL3-promoter vector (Promega, WI, USA). Primer sequences for cloning pre-miRNA and N-ras 3'UTR sequences are as follows.

Pre-miR-1 for:TTGCGGCCGCAA GCTTGGGACACATACTTCTT

Pre-miR-1 rev: GGTTTAAACC GCCTGAAATACATACTTCT

Pre-miR-214 for: TTGCGGCCGCAA GGCCTGGCTGGACAGAGTT

Pre-miR-214 rev: GGTTTAAACC AGGCTGGGTTGTCATGTGACT

 FL-for: CTATGAAAATTTCAAAACAGT

 FL-rev: GAATATAAGAATTATGACTAAGCC

 S1-for: CTTCCACAGCACAAACAC

 S1-rev: AACAAACCAAACAGCAAT

 S2-for: GTTTAGTCTTTCACCATCC

 S2-rev: GAAGCAGAACGCACCATT

 S3-for: ATATCAGTACTTGAGGATTCAACCGT

 S3-rev: ATTATGACTAAGCCAAGAA

### RNA oligonucleotides and transfection

MicroRNA mimics and inhibitors were purchased from Dharmacon, Inc. For transient transfection of cells in 6-well plates, 200 μM mimics or inhibitor were used with Oligofectamine (Invitrogen) as the delivery agent. Cell numbers were counted every 24 hour after trypsinization using a hemacytometer. For generating the stable cell lines, P2GM, P2GM-miR-1(P-1) or P2GM-miR-214(P-214) plasmids were transfected into RD cells using Lipofectamie according to the manufacturer's procedure (Invitrogen). Transfected cells were selected with 10μg/ml of puromycin for 2 weeks, and resistant cells were pooled and chosen for subsequent experiments.

Sequences of miR214 inhibitor or mimics as well as nonspecific control are as follows.

Negativecontrol:UUCUCCGAACGUGUCACGUTT

MiR-214 mimic: ACAGGUAGUCUGAACACUGGGUU

InhibitorNC :CAGUACUUUUGUGUAGUACAA

MiR-214 inhibitor:ACUGCCUGUCUGUGCCUGCUGU

### Reverse transcription and RT-PCR

Total RNAs from cultured cells, human skeletal muscles, and tumors were extracted using the RNAiso reagent and the cDNA was synthesized in reverse transcription reactions using the PrimeScript RT reagent kit (from a TAKARA distributor, China). SYBR green real-time qPCRs were carried out on a ABI7500 Real-Time PCR system. TaqMan quantitative real-time PCR detection of microRNAs was done using the microRNA assay kit (Applied Biosystems, USA), and the U6 snRNA was used for normalization. The cycling condition for both SYBR green and Taqman real-time PCR was 95 °C for 5 minutes, followed by 40 amplification cycles of 95 °C, 15 second and 60 °C, 1 minute. For each data point, triplicate reactions were carried out and the experiment was repeated three times to assess the statistical significance. RT-PCR primer sequences are listed in the additional Materials and Methods.

### MTT assay

To measure cell growth, 24 hours after transfection with miRNA mimics, cells were trypsinized and reseeded in a 96-well plate. 24 hours later, 100 μl MTT (5 mg/ml) was added to each well and the cells were incubated for additional 4 hours. After discarding the MTT-containing medium, 100 μl of DMSO was added and the absorbance at 490 nm was measured using a multi-well spectrophotometer.

### Cell cycle and apoptosis assays

To assess cell cycle properties, cells were transfected with microRNA mimics or the ns control using oligofectamine in 6-well plates. 48 hours later, transfected cells were collected and incubated with 100 μg/ml propidium iodide (PI) and 0.5 μg/ml RNase A for 30 minutes at the room temperature before subjecting to FACS analysis. PI-negative viable cells were gated out during the cell cycle analysis. To assess apoptosis, 24 hours after transfection the cells were transferred to DMEM supplemented with 0.2% FBS or maintained in the 10% FBS medium and incubated for 72 hours. The apoptotic cells were analyzed using the Annexin V-PI apoptosis detection kit according to the manufacturer's instruction (BD, USA).

### Edu incorporation assay and Ki67 staining

To measure cell growth, RD cells were transfected with microRNA mimics in 24-well plates. 48 hours after transfection, 20 μM EdU was added for 1 hour, and MEFs from wt and miR-214 knockout mice were incubated with 20 μM EdU for 4 hours or 6 hours. The cells were then fixed in 3.7% formaldehyde. After washing with PBS and permeablization, 500 μl Click-iT reaction cocktail (430 μl 1xClick-iT reaction buffer, 20 μl CuSO_4_, 1.2 μl Alexa Fluor® azide, and 50 μl reaction buffer) was added to each well and incubated for 45 minutes at the room temperature in the dark. The cells were counter-stained with DAPI for nuclei and visualized under an inverted fluorescence microscope. Images were processed with Image J and the percentage of EdU incorporation was calculated based on the numbers of EdU positive (red) and total (DAPI) cells. Ki67 staining was carried out using anti-Ki67 monoclonal antibody (Abcam) at 4°C overnight, and the fluorescence images were processed as above.

Western analyses and luciferase assays 30 μg total protein from miRNA mimics transfected cells were loaded onto each lane of a 12% SDS-polyacrylamide gel. Anti-N-ras, anti-Myogenin, and anti-p21 (all from Santa Cruz, USA) and anti-GAPDH (Kangchen, China) were used as the primary antibodies. Firefly reporter luciferase and the co-transfected renilla luciferase activities were measured 48 hours after transfection using the Dual-Luciferase Reporter System (Promega).

### Tissue samples

Human normal lung and cancer tissues, prostatic tumors and their matching adjacent non-tumor tissues were obtained from the Nanjing Medical University No.1 affiliated hospital. Written consent forms were obtained from all patients before collection. All tissue samples were histologically confirmed with hemotoxylin-eosin staining. This study was approved by the Research Ethics Committee of Nanjing Medical University.

### Statistical analyses

Statistical analyses were performed in the GraphPad Prism 5.0 environment. Data were analyzed by one-way ANOVA and nonparametric analyses. All pairs of columns were compared bars denote mean ± SD. P values less than 0.05 were considered statistically significant. * = p<0.05, ** = p<0.01, and *** = p<0.001.

## SUPPLEMENTARY FIGURES AND METHODS


