# Supplementary material for: MiR-214 and N-ras regulatory loop suppresses rhabdomyosarcoma cell growth and xenograft tumorigenesis
Source: Oncotarget. 2014 Mar 25;5(8):2161–75. doi: 10.18632/oncotarget.1855 (PMC4039153; doi:10.18632/oncotarget.1855)
Supplement: Supplementary file 1 [file oncotarget-05-2161-s001.pdf]

MiR-214 and N-ras regulatory loop suppresses rhabdomyosarcoma cell growth and xenograft tumorigenesis

Additional materials and methods

Animal strains and miR-214 gene targeting C57BL/6J, EIIa-cre, and BALB/C nude mice were purchased from the Model Animal Research Center (MARC) of Nanjing University. Gene-targeting and animal husbandry (Animal Protocol No. NRCMM004) were carried out under the supervision of Institutional Animal Care and Use Committee of MARC in accordance the laboratory animal care and use regulations (National Research Council, 2011). Mice were maintained on a normal 12h/12h light and dark cycle with regular mouse chow and water ad libitum in an AAALAC International accredited specific pathogen-free facility. IVC were used for all mice within barrier facility.

Southern blot and PCR genotyping primers Genomic DNA was isolated from ES cells and clipped tails by proteinase K digestion in 20 mM Tris–HCl (pH 7.5), 100 mM NaCl, 10 mM EDTA, and 0.5% SDS, 37 °C for 1 hr, followed by NaCl extraction and ethanol precipitation. For Southern blot analyses, 20 μg genomic DNA was digested with BamHI and NotI, separated in a 0.8% agarose gel, and transferred to a nylon membrane (Hybond N+, GE Healthcare). Blots were prehybridized in HB (4×SSC, 0.5% SDS, 5×Denhardt's solution, 100 μg/ml denatured salmon testis DNA) at 67 °C for 4 hr, and then hybridized to denatured ^{32}P-labelled DNA probe at 67 °C for 20 hr. Genotyping PCR primer sequences are as follows.

Loxp-for:GATTCATGGTCTCACCCTGCTTG

Loxp-rev:CAAGACCTCTGCATCTCTTAATAGTG

Del-for:CTGTGTTTAAGGTCCTACTCTG

Del-rev:CATCCAACTGAAGCCAAA

CRE-for:TGCCACGACCAAGTGACAGCAATG

CRE-rev:AGAGACGGAAATCCATCGCTCG

RT-PCR primer sequences were as follows.

miR-214-RT:GTCGTATCCAGTGCAGGGTCCGAGGTATTCGCACTGGATACGAC CTGCCT

miR-214-For: GTCTTAACAGCAGGCACAGAC

miR-214-Rev: GTGCAGGGTCCGAGGT

U6-RT：CGTTCACGAATTTGCGTGTCAT

U6-For：CGCTTCACGAATTTGCGTGTCAT

U6-Rev: GCTTCGGCAGCACATATACTAAAAT

BNP-For：CTGCTGGAGCTGATAAGAGA

BNP-Rev: TGCCCAAAGCAGCTTGAGAT

βMHC-For: GTGC-CAAGGGCCTGAATGAG

βMHC-Rev: GCAAAGGCTCCAGGTCTGA

18S-For: GTAACCCGTTGAACCCCATT

18S-Rev: CCATCCAATCGGTAGTAGCG

MyoG-For: GGGGATCATCTGCTCACGG

MyoG-Rev: TCAGTTGGGCATGGTTTCAT

MHC-For: TACTTCTACGACGGCTCCTG

MHC-Rev: GTGGTGGACTTCCTCTTGC

human Nras-For: AACAAGCCCACGAACT

human Nras-Rev: TGGCAATCCCATACAA

mouse Nras-For: CCTTGACCCGTTTGAC

mouse Nras-Rev: AACCACCTACATACCTACAT

mouse Ezh2-For: GACACTCCTCCAAGAAAGAAGAA

mouse Ezh2-Rev: GGATGGTCACAGGGTTGATAG

human Ezh2-For: AGTCCTGAAGAAGAGACATTCC

human Ezh2-Rev: GAAGCCTAGCTCCTTGTACTT

mouse Pten-For: CCCACCACAGCTAGAACTTATC

mouse Pten-Rev: CGTCCTTTCCCAGCTTTACA

mouse Dnm3os-For: TTCGCACTCTTCAACACC

mouse Dnm3os-Rev: CATTCGGAGCATCTCATCT

mouse Dnm3-For: AGAAAGCCTGTCCTGGTAT

mouseDnm3-Rev: CTCGGTGTTGAAGAGTGC

Colony formation assays For anchorage-dependent colony formation assay, approximately 500 stable RD cells carrying P2GM-1, P2GM-214, or the parental P2GM vector were seeded in 60 mm petri dishes in duplicates. The cells were maintained in complete growth media and allowed to grow for 14 days. The petri dishes were then fixed in cold methanol, stained with 0.25% crystal violet in water, and the number of foci containing over 50 cells was counted. For anchorage-independent colony formation assay, 4 mL 0.7% low-melting agarose in DMEM plus 10% FBS was added in a 60 mm petri dish to prepare for the under layer. 1×10^5 RD cells under different treatments were trypsinized and mixed in 0.4% low-melting agarose for making the top layer. The cells were maintained in complete growth media for 3 weeks until visible foci (containing over

50 cells) appear. The petri dishes were then fixed in cold methanol, stained with 0.25% crystal violet in water, and the number of foci was counted under the microscope.

Xenograft tumor models Stable RD cells carrying P2GM-1, P2GM-214, or the parental P2GM vector were trypsinized, counted, and resuspended at $6.7x10^7$ cells/ml in PBS. 150 μl of the cell suspension was injected into each side of the lower back of a female athymic nude mouse (BALB/c-nu/nu) at 6 weeks of age. The maximal (a) and minimal (b) diameters of each tumor were measured every week with a precision caliper and the tumor volume was calculated based on the modified ellipsoid formula V (mm^3) = $1/2ab^2$. The tumor-bearing mice were euthanized 8 weeks after the injection.

Immunohistochemistry staining Paraffin-embedded tissue blocks were cut into 5 μm thick sections and mounted on glass slides. The leiomyosarcoma and rhabdomyosarcoma tumor array was purchased from Biomax, Inc (Cat.# SO751, Gaithersburg, MD). This tissue microarray contains duplicated sections of 36 leiomyosarcomas and 36 rhabdomyosarcomas, plus 1 section each of normal smooth, skeletal, and cardiac muscle tissue as controls. Rhabdomyosarcma sample include embryonic (ERMS), alveolar (ARMS), pleomorphic (PRMS), and spindle cell (SRMS) subtypes. The sections were deparaffinized in xylene and rehydrated with graded ethanol washes. The slides were incubated with goat anti-N-ras antibody (Abcam) at 4℃ overnight, washed, and a biotinylated anti-goat secondary antibody was added for 30 minutes. The sections were subsequently reacted with a streptavidin-peroxidase conjugate and 3’-3’-

diaminobenzidine. A negative control slide was prepared following the same procedure, except that the anti-N-ras was replaced by the normal goat IgG.

Cardiotoxin injection 6-week old wild type or miR-214$^{-/-}$ male mice were anesthetized by intraperitoneal injection of 3.6% chloral hydrate at 80 μl/10g body weight. Tibilais anterior (TA) muscle from hind limbs were shaved to expose the muscle group and wiped with 70% ethanol. 50 μl of cardiotoxin (CTX) (Sigma, USA) at 20 μM was injected into the belly of TA, and the mice were allowed to recover on a heating pad for 1 hour. TA were harvested at days 1, 2, 3, 5, 10, and 14 following the injection, and fixed in 4% paraformaldehyde for histological analysis.

Cardiac hypertrophy model by transverse aortic constriction (TAC) 6-8 weeks old wild type or miR-214$^{-/-}$ male mice (body weight ranges from 22-25 grams) were used for establishing cardiac hypertrophy models as described (deAlmeida et al., 2010). Briefly, the animals were anesthetized on a rodent ventilator. The heart was exposed through thoracotomy on the left side in the third intercostal space. After the transverse aorta was isolated from carotid arteries, a 7-0 silk suture was drawn under the transverse aorta and tied against a 26-gauge needle that was promptly removed. For age-matched sham operations, the identical surgical procedure was performed but the aorta was not tied by the suture. Two weeks after TAC, the heart was harvested and the ratios of heart weight/body weight (HW/BW) and left ventricular weight/tibia length (LVW/TL) were calculated. The heart was snap-frozen in liquid nitrogen for RNA and protein isolation or fixed in 4% paraformaldehyde for histological analysis.

Supplementary Figure Legends

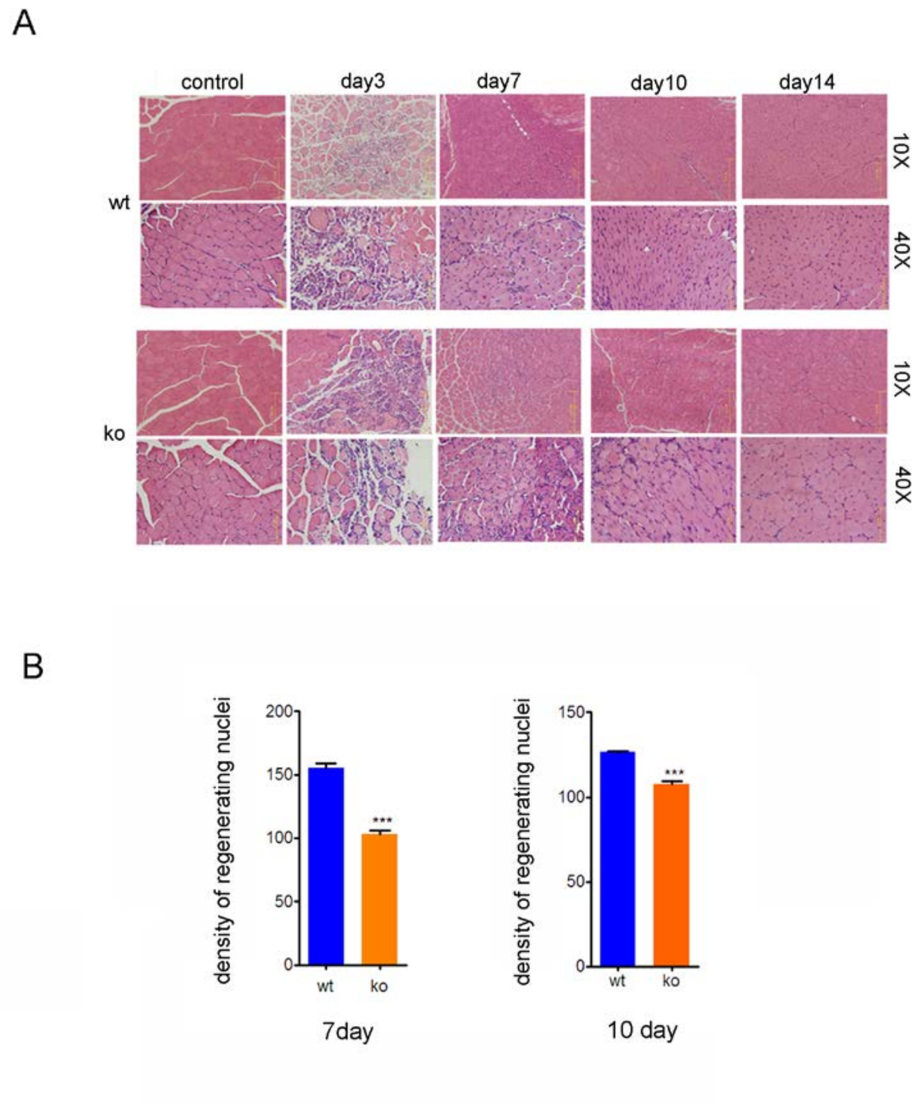

sFig.1 H&E staining of cardiotoxin III-induced tibia muscle injury and regenerative repair sites. We injected tibia calves of miR-214$^{-/-}$ and the littermate WT control mice with cardiotoxin III and analyzed myofiber regeneration at various time points. Three days after injection, the animals exhibited extensive myofiber degeneration and small mononucleated cells, including proliferation myoblasts, macrophages, and neutrophils,

accumulated at the side of injury. Seven days after the injection, most of the damaged myofibers in WT mice were cleared and replaced by newly formed myofibers containing centralized nuclei. In contrast, abundant inflammatory cells and damaged myofibers were still present in miR-214$^{-/-}$ mice. Ten days after the injection, injury site in WT mice were filled with newly formed multinucleated myotubes. At this time point, damaged myofibers in miR-214$^{-/-}$ mice began to be replaced by newly formed myofibers. Fourteen days after the injection, normal muscle architecture was restored in both WT and miR-214$^{-/-}$ mice, suggesting that loss of miR-214 may delay but not completely prevent regeneration. (B) Density of regenerating (centralized) nuclei in the damaged area at 7 or 10 days post injury.

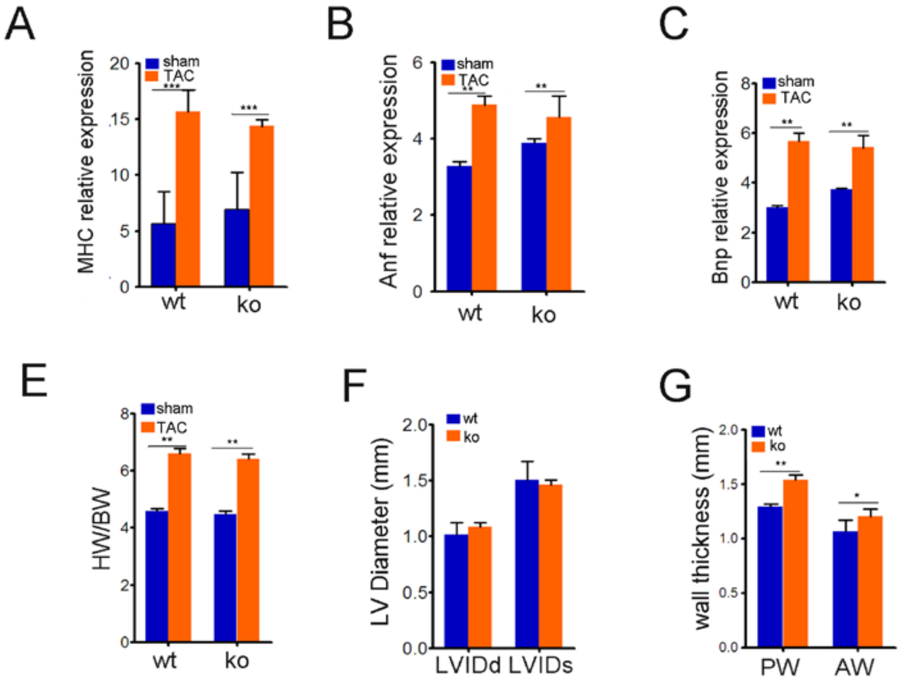

sFig.2 Comparison of thoracic aortic constriction between miR-214$^{-/-}$ and the littermate WT control mice Thoracic aortic constriction (TAC), which recapitulates aortic stenosis developed as the result of pressure-overload-induced left ventricular hypertrophy, was performed on miR-214$^{-/-}$ and the littermate WT control mice. The mice were sacrificed 2 weeks following the surgical procedure, significantly increased the heart to body weight ratio, but no different between WT and miR-214-KO mice. Response to TAC β-MHC and other stress-responsive genes, such as those encoding the natriuretic peptides ANF and BNP, were strongly induced, but no different between WT and miR-214-KO. Functional analysis by echocardiography after TAC include the left ventricular walls (anterior wall in systole and posterior wall in systole) and left

ventricular diameter (left ventricular internal diameter in diastole and systole) also indicated no differences between the two genotypes .

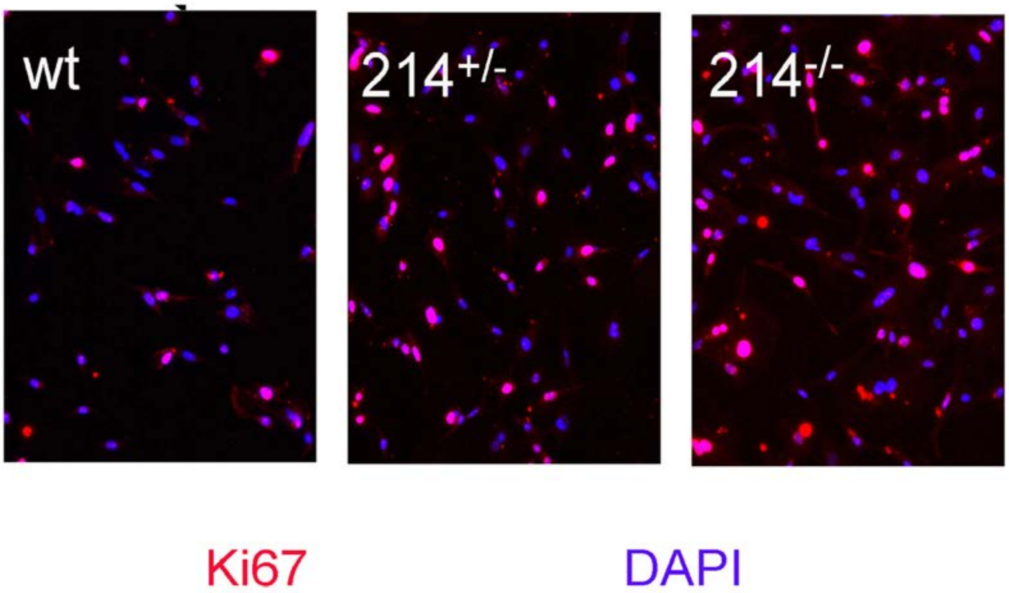

sFig.3 Images of Ki67 immunofluorescence staining for Fig.1F.

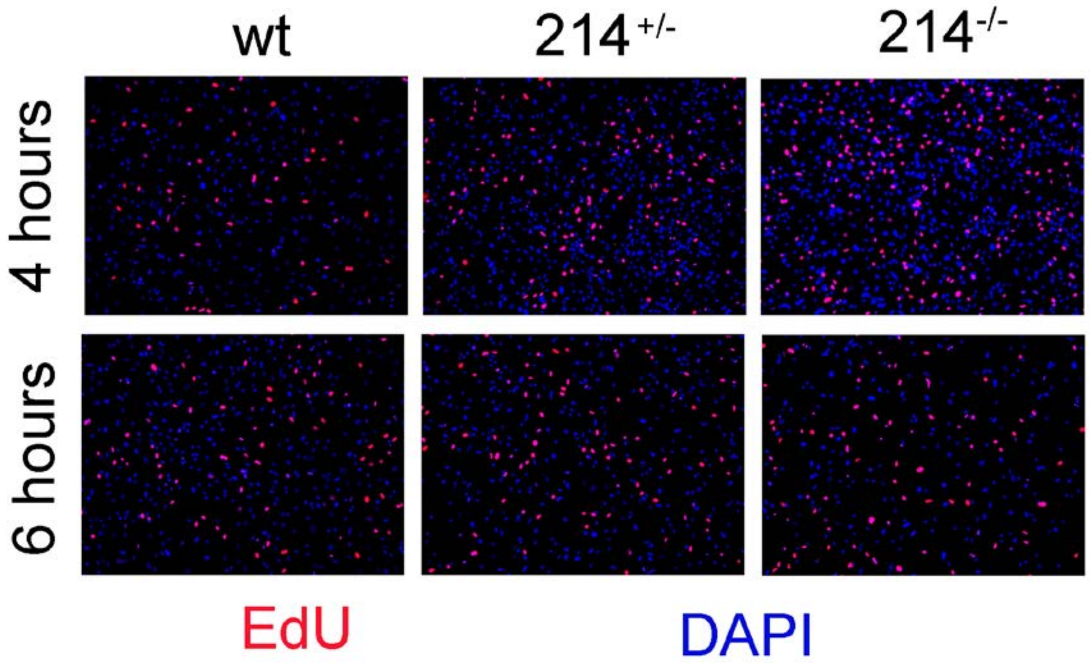

sFig.4 Images of EdU incorporation assay for Fig.1G.

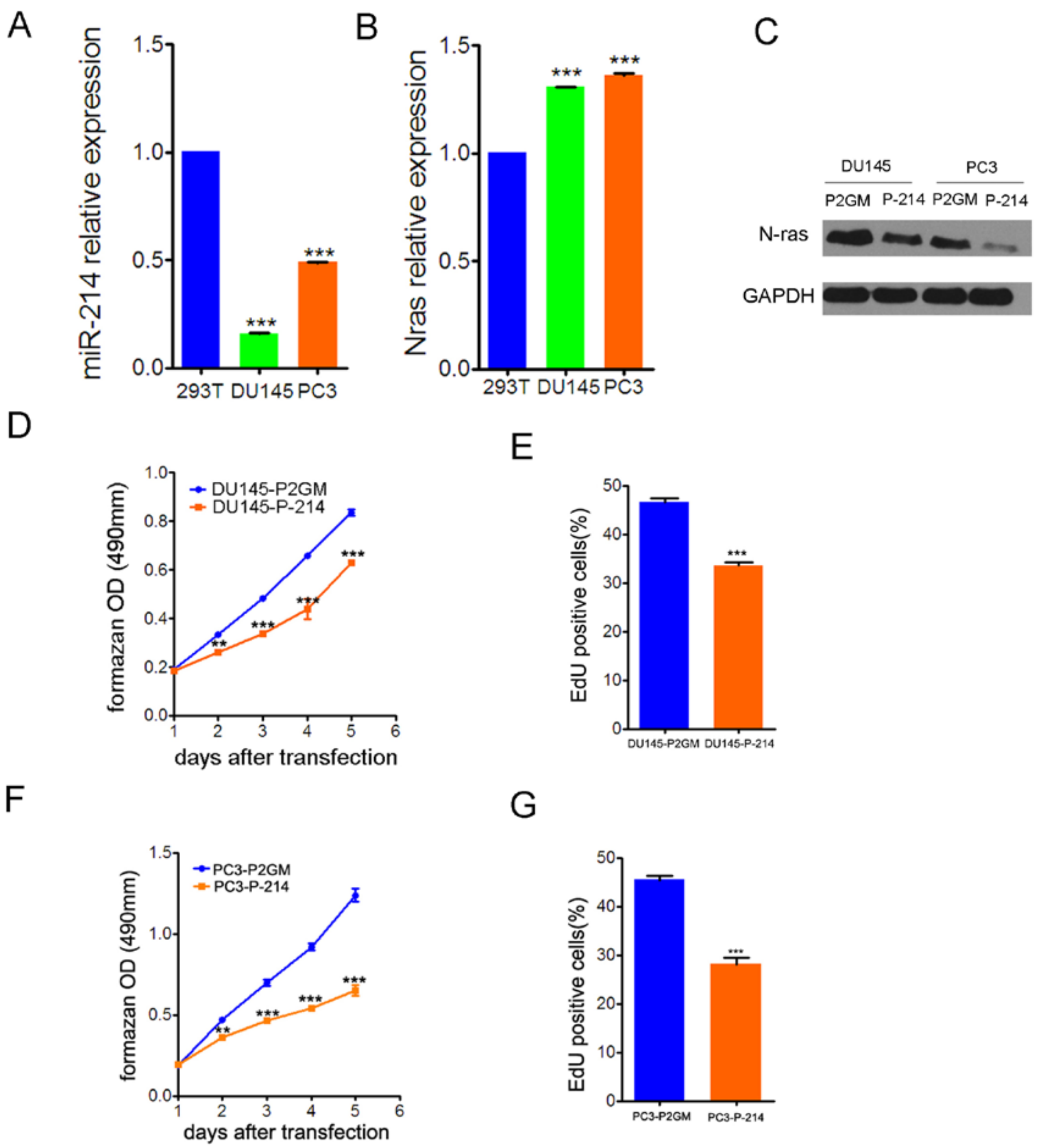

sFig.5 miR-214 suppresses the proliferation of prostate cancer cells DU-145 and PC-3 (A) RT-qPCR quantification of miR-214 expression. The data were compiled from three rounds of experiments and are presented as mean ± standard deviation. (B) RT-qPCR and (C) Western blot analyses of N-ras in DU145 and PC3 cells transfected with P2GM empty vector and P-214. (D) MTT and (E) EdU incorporation assays for measuring growth of DU-145 cells. (F) MTT and (G) EdU incorporation assays for

measuring growth of PC-3 cells. The cells were transiently transfected with P-214 or the P2GM empty vector. Data were presented as mean ± standard deviation. Asterisks denote P values calculated based on t-test. * = $p<0.05$, ** = $p<0.01$, and *** = $p<0.001$.

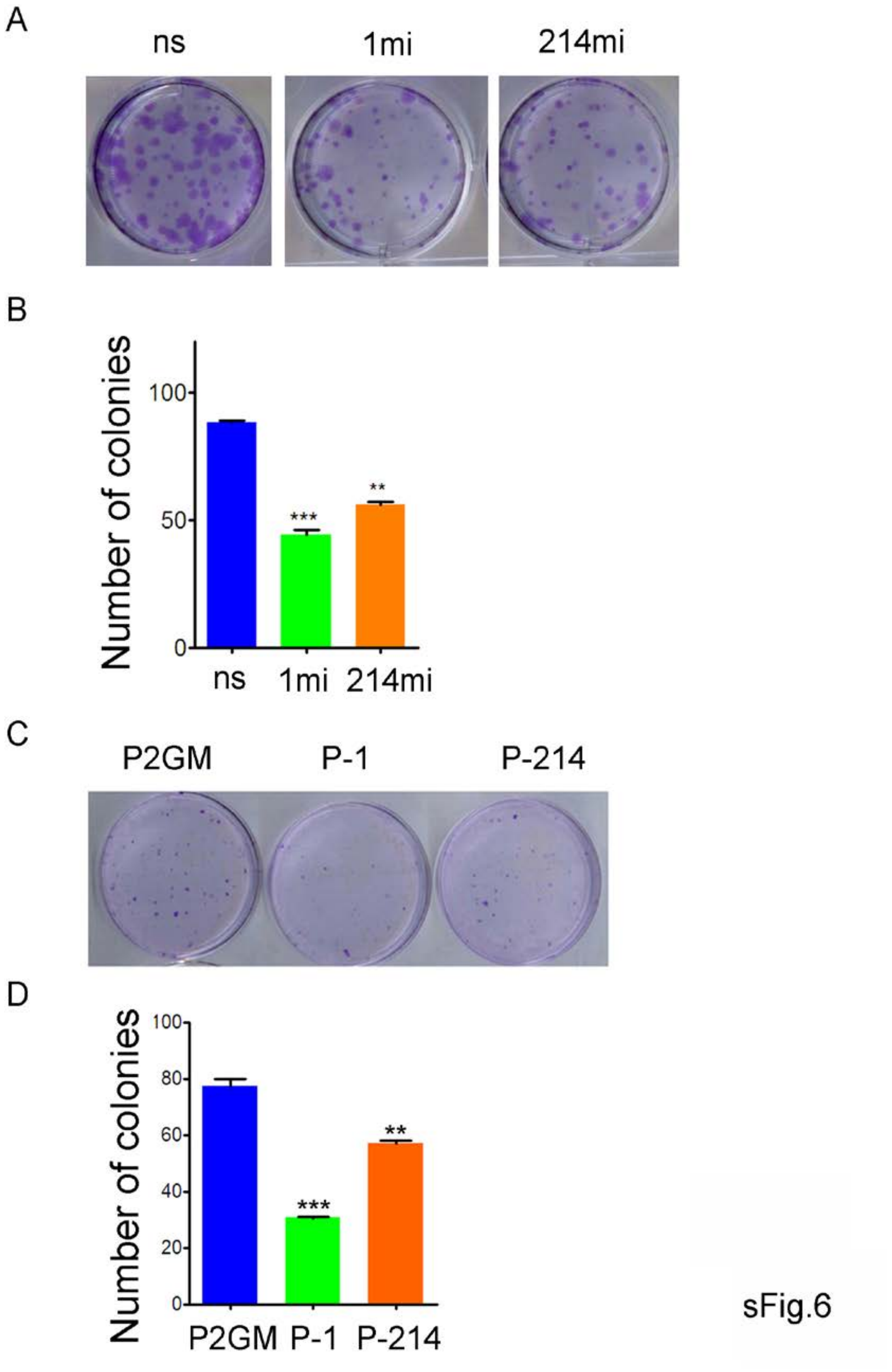
A
ns
1mi
214mi
B
Number of colonies
100
50
0
***
**
ns
1mi
214mi
C
P2GM
P-1
P-214
D
Number of colonies
100
80
60
40
20
0
***
**
P2GM
P-1
P-214
sFig.6

sFi

g.6 Anchorage-dependent and –independent colony formation assays (A) 500 RD cells transiently transfected with ns, miR-1mi, and miR-214mi were cultured in 60 mm petri dishes in regular culture medium supplemented with 10 μg/ml puromycin for 14 days. The plates were then fixed and stained with crystal violet for colony counting. (B) Quantification of (A). (C) For anchorage-independent growth assays, $1x10^5$ stable RD cell were cultured in the top agarose in 60 mm petri dishes in regular culture medium supplemented with 10 μg/ml puromycin for three weeks. The plates were then fixed and stained with crystal violet for colony counting. (D) Quantification of (C).

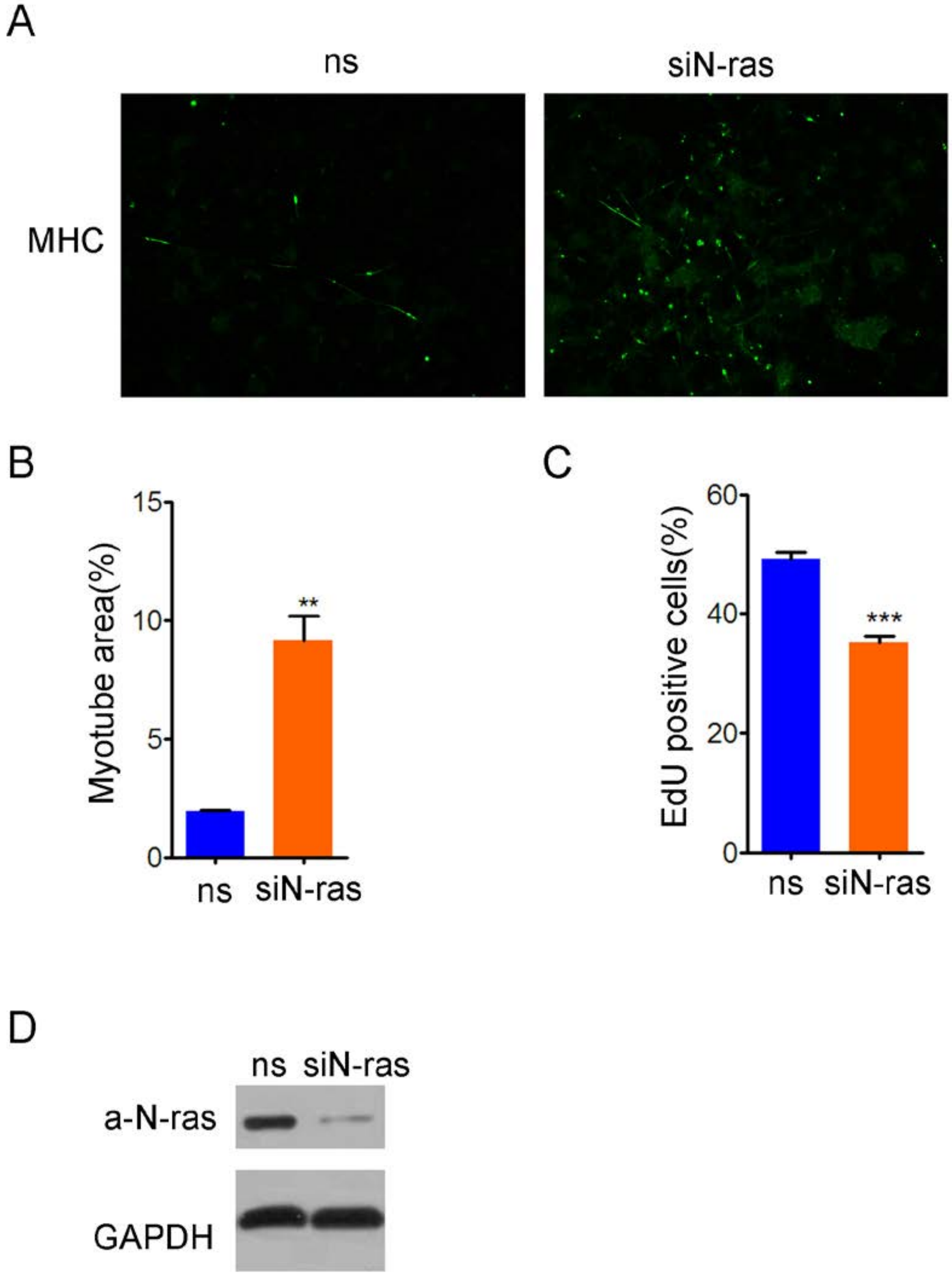

sFig.7 Human N-ras plays active regulatory roles in human RMS cells (A) IF staining of MHC and (B) quantification of myotube areas following myogenic differentiation of RD cells transfected with siN-ras or the nonsilencing control siRNAs. (C) Quantification

of Edu incorporation in RD cells transfected with ns or siN-ras. (D) Western blot analysis showing the N-ras level following transfection of siN-ras.
